# 4-1BB/4-1BBL Interaction Promotes Obesity-Induced Adipose Inflammation by Triggering Bidirectional Inflammatory Signaling in Adipocytes/Macrophages

**DOI:** 10.1155/2012/972629

**Published:** 2012-12-17

**Authors:** Thai Hien Tu, Chu-Sook Kim, Tsuyoshi Goto, Teruo Kawada, Byung-Sam Kim, Rina Yu

**Affiliations:** ^1^Department of Food Science and Nutrition, University of Ulsan, Ulsan 680-749, Republic of Korea; ^2^Graduate School of Agriculture, Kyoto University, Uji, Kyoto 611-0011, Japan; ^3^Department of Biological Science, University of Ulsan, Ulsan 689-749, Republic of Korea

## Abstract

Obesity-induced adipose inflammation is characterized by recruitment of macrophages to adipose tissue and release of inflammatory cytokines. 4-1BB, a costimulatory receptor, modulates inflammatory processes through interaction with its ligand 4-1BBL on immune cell surfaces. In this study, we examined whether a 4-1BB/4-1BBL interaction between adipocytes and macrophages participates in obesity-induced adipose inflammation. We found that 4-1BB was expressed on adipocytes and was upregulated by obesity-related factors, which also enhanced 4-1BBL expression on macrophages. 4-1BB and/or 4-1BBL agonists, respectively, activated inflammatory signaling molecules (MAPK/I**κ**B**α** and MAPK/Akt) in adipocytes and macrophages and enhanced the release of inflammatory cytokines (MCP-1, TNF-**α**, and IL-6). Moreover, disruption of the 4-1BB/4-1BBL interaction decreased the release of inflammatory cytokines from contact cocultured adipocytes/macrophages. These findings indicate that 4-1BB/4-1BBL-mediated bidirectional signaling in adipocytes/macrophages promotes adipose inflammation. 4-1BB and 4-1BBL may be useful targets for protection against obesity-induced adipose inflammation.

## 1. Introduction

Obesity-induced inflammation is considered to be a potential cause of metabolic disorders such as insulin resistance, type 2 diabetes, and cardiovascular diseases [1–3]. Adipose tissue actively participates in obesity-induced inflammation through recruitment of macrophages and T cells and release of inflammatory cytokines (monocyte chemotactic protein-1, MCP-1; tumor necrosis factor alpha, TNF-*α*; interleukin-6, IL-6) which modulate adipocyte differentiation, metabolism, and local/systemic inflammatory responses, causing undesirable metabolic imbalances [2, 4]. Interestingly, recent studies have shown that direct contact coculture of adipocytes and macrophages results in markedly elevated release of inflammatory cytokines [5–7], indicating that interaction between cell surface molecules on these cells is important for promoting their inflammatory responses. 

4-1BB (also known as CD137 and TNFRSF9) is a classic example of a costimulatory molecule, and a well-known inflammatory receptor that is expressed by activated T cells at sites of inflammation [8]. Stimulation of 4-1BB on T cells leads to cell expansion, cytokine production, and development of cytolytic effector functions [9]. 4-1BB ligand (4-1BBL, also known as CD137L and TNFSF9) is highly expressed by most immune and many nonimmune cells and can receive and transmit reverse signals into cells such as macrophages [10]. Accumulating evidence shows that bidirectional cell surface 4-1BB/4-1BBL interactions in immune cells are critical in initiating and modulating various inflammatory responses (e.g., rheumatoid arthritis, autoimmune myocarditis, and hematological malignancies) [11–13]. Moreover, 4-1BB/4-1BBL-mediated interactions also occur between immune and nonimmune cells, again influencing inflammatory responses. For example, interaction between 4-1BB and 4-1BBL on endothelial cells and macrophages is involved in vascular inflammation [14, 15], and interaction between the two molecules on epithelial cells and natural killer cells is involved in renal ischemia-reperfusion injury [16]. We previously showed that expression of 4-1BB and 4-1BBL was upregulated in adipose tissue that was inflamed due to obesity, and that ablation of 4-1BB reduced adipose inflammation [17]. Hence, we hypothesized that interaction between 4-1BB and 4-1BBL on adipose cells and immune cells such as macrophages plays a role in adipose inflammation in obesity. 

In this study, we show for the first time that 4-1BB is expressed on adipocytes and is upregulated by obesity-related factors, and we demonstrate that 4-1BB/4-1BBL-mediated bidirectional signaling in adipocytes/macrophages plays a crucial role in initiating and promoting the obesity-induced adipose inflammatory cascade.

## 2. Materials and Methods

### 2.1. Animals

C57BL/6 mice (male, 8 weeks old) (Orient Ltd., Busan, Korea) were fed a high-fat diet (HFD, 60% of calories from fat (Research Diets Inc., New Brunswick, NJ, USA); obese mice) or a low fat diet (LFD; 10% of calories from fat (Research Diets); nonobese mice) for 9 weeks. All animal experiments were approved by the animal ethics committee of the University of Ulsan and conformed to National Institutes of Health guidelines.

### 2.2. Antibodies

Nude mice were primed with pristane and injected intraperitoneally with a subcloned hybridoma producing an agonistic monoclonal antibody (Ab) against 4-1BB (3E1) to induce ascite formation [18]. The monoclonal Ab was purified from the ascites fluid by affinity column chromatography with protein G-Sepharose (Sigma-Aldrich). Recombinant 4-1BB Fc (r4-1BB Fc) was purchased from Adipogen (Seoul, Korea). Antagonistic monoclonal Ab against 4-1BBL (TKS-1) was purchased from e-Bioscience (San Diego, CA, USA). Rat immunoglobulin G (Rat IgG) and human IgG1 were purchased from Sigma-Aldrich (St. Louis, MO, USA) and used as control.

### 2.3. Cell Cultures and Treatments

The murine macrophage cell line Raw264.7 was obtained from the Korean Cell Line Bank (KCLB40071, Seoul, Korea). This cell line was maintained in RPMI1640 (Gibco BRL, NY, USA) containing 10% (vol/vol) FBS (fetal bovine serum) (Gibco BRL, NY, USA) and incubated at 37°C in humidified 5% CO_2_. 3T3-L1 preadipocytes were maintained in DMEM (Dulbecco's Modified Eagle Medium) high glucose (Gibco BRL, NY, USA) containing 10% FBS. Confluent 3T3-L1 preadipocytes (day 0) were incubated in DMEM containing 10 *μ*g/mL insulin (Sigma-Aldrich), 0.25 *μ*M DEX (dexamethasone, Sigma-Aldrich), 0.5 mM IBMX (3-isobutyl-1-methyl-xanthine, Sigma-Aldrich), and 10% FBS for 2 days. Briefly, 3T3-L1 cells were differentiated into mature adipocytes by incubation in DMEM with 10% FBS and 5 *μ*g/mL insulin for 2 days. Mature adipocytes were maintained in this medium, and the culture medium was replaced with fresh medium every 2 days. Free fatty acid (FFA, palmitic acid mixture, Sigma-Aldrich) was dissolved in ethanol containing bovine serum albumin (BSA, 25 *μ*M) and conjugated with BSA at a 10 : 1 molar ratio before use. 3T3-L1 adipocytes (3 × 10^5^ cells/well) or Raw264.7 macrophages (3 × 10^5^ cells/well) in 24-well plates, were treated with obesity-related factors (palmitic acid : pal, lipopolysaccharide : LPS) for 24 h or 4 h, respectively. To stimulate 4-1BB on adipocytes, 3T3-L1 adipocytes on 24-well plates were incubated with agonistic 4-1BB Ab (3E1, 1 *μ*g/mL) or rat IgG for 48 h in serum-free medium. To immobilize r4-1BB Fc or human IgG1 on culture plates, r4-1BB Fc or human IgG1 were incubated in 24-well plates at 37°C for 1 h in a CO_2_ incubator and the wells were rinsed with phosphate buffered saline (PBS). The plates were then incubated with RPMI (10% FBS) at 37°C for 1 h in a CO_2_ incubator and the wells were rinsed with PBS. Raw264.7 macrophages were incubated at 5 × 10^5^ cells/well in 24-well flat-bottomed plates precoated with 100 ng/mL r4-1BB Fc or human IgG1 for 24 h.

### 2.4. Isolation of Stromal Vascular Cells from Adipose Tissue

To isolate the stromal vascular fraction (SVF) of adipose tissue, epididymial fat pads of C57BL/6 mice (male, 8 weeks old) were minced and digested for 30 minutes at 37°C with type 2 collagenase (1 mg/mL; Sigma-Aldrich) in DMEM (pH 7.4). The resulting suspensions were centrifuged at 500 g for 5 minutes. The pellets were resuspended in erythrocyte lysis buffer, and the suspensions incubated at room temperature for 3 minutes, then centrifuged at 500 g for 5 minutes. After washing in DMEM, the suspensions were passed through sterile 100 *μ*m nylon meshes (SPL Lifescience, Pocheon, Korea). The filtrated cells were transferred to 100 mm^2^ dishes contained DMEM supplemented with 10% FBS and 0.4% Fungizone and maintained in an incubator at 37°C in 5% CO_2_. Cells were allowed to attach and floating cells were removed by aspiration and the culture media was replenished for every day. The SVF cells were collected after 2 day. The SVF cells were plated at 5 × 10^5^ cell/well in 24-well plates. The confluent SVF-derived preadipocytes were differentiated into adipocytes by treatment with DMEM containing 10 *μ*g/mL insulin (Sigma-Aldrich), 0.25 *μ*M DEX (Sigma-Aldrich), 0.5 mM IBMX (Sigma-Aldrich), and 10% FBS for 2 days. Mature adipocytes were maintained in the culture medium that was replaced with fresh medium every 2 days. To detect 4-1BB and 4-1BBL expression on SVF-derived adipocytes exposed to obese factors, these cells incubated with palmitic acid 250 *μ*M, LPS 100 ng/mL for 24 h. 

### 2.5. Isolation of Peritoneal Macrophages

C57BL/6 mice (male, 8 weeks old) were intraperitoneally injected with 3 ml of 3% thioglycollate broth (Difco, Detroit, MI, USA) 4 days before being killed. Peritoneal macrophages were collected by centrifugation in MEM media (Minimum Essential Medium, Gibco) and the resulting pellet was washed and resuspended in culture medium MEM with 10% FBS. The peritoneal macrophages were purified by adherence to tissue culture plates for 2 hours [19]. To detect 4-1BB and 4-1BBL expression on peritoneal macrophages exposed to obese factors, these cells incubated with palmitic acid 250 *μ*M, LPS 100 ng/mL for 4 h.

### 2.6. Coculture of Adipocytes and Macrophages

3T3-L1 adipocytes were cultured and differentiated for 6 days. Coculture of adipocytes and macrophages was performed by two methods: direct contact coculture and transwell coculture. In the direct contact system, Raw264.7 macrophages (3 × 10^5^ cells: 50% macrophages, 3 × 10^4^ cells: 10% macrophages) or peritoneal macrophages (3 × 10^5^ cells) were placed in 24-well plates containing 3T3-L1 adipocytes (3 × 10^5^ cells). The cells were cultured for 24 h in contact with each other and harvested. As a control, adipocytes and macrophages were also cultured separately, with cell numbers per well equal to those in the contact system and mixed after harvest. In the trans-well system, cells were cocultured using trans-well inserts with a 0.4 *μ*m porous membrane (Corning, NY, USA) to separate adipocytes (3 × 10^5^ cells, lower well) from macrophages (3 × 10^5^ cells, upper well). After incubation for 4 h, 8 h, and 12 h, the supernatants were harvested.

### 2.7. Measurement of Cytokine Levels

Cytokine levels in culture supernatants were measured using enzyme-linked immunosorbent assays (ELISA). The assays were conducted using OptEIA mouse TNF*α*, a mouse MCP-1 set (BD Bioscience Pharmingen, CA, USA) and a mouse IL-6 and adiponectin set (R&D Systems, Minneapolis, MN, USA), and IL-10 kit (R&D Systems, Minneapolis, MN, USA). Values for cytokine levels were derived from standard curves using the curve-fitting program SOFTmax (Molecular Devices, Sunnyvale, CA, USA).

### 2.8. Quantitative Real-Time PCR (qRT-PCR)

Total RNA extracted from cultured cells was reverse transcribed to generate cDNA using M-MLV reverse transcriptase (Promega, Madison, WI, USA). Real-time PCR amplification of the cDNA was performed in duplicate with a SYBR premix Ex Taq kit (TaKaRa Bio Inc., Foster, CA, USA) using a Thermal Cycler Dice (TaKaRa Bio Inc., Japan). All reactions were performed by the same procedure: initial denaturation at 95°C for 10 s, followed by 45 cycles of 95°C for 5 s and 60°C for 30 s. All values for genes of interest were normalized to values for housekeeping genes (36B4 for adipocytes; *β*-actin for macrophages and cocultures). Mouse primer sequences used are shown in Table 1.

Data were analyzed using Thermal Cycler Dice Real Time System Software (Takara Bio, Inc.). Relative standard curves were generated by plotting the cycle threshold (Ct) values. Base on the Ct values obtained from each sample, the relative amounts of target genes were calculated using the standards curves with software provided by Takara Thermal Cycler Dice Real Time System.

### 2.9. Separation of Adipocytes and Macrophages

Cocultured 3T3-L1 adipocytes and Raw264.7 macrophages in equal numbers (as described earlier) were separated following the manufacturer's protocol, using the CD11b MicroBeads system (MACS; Miltenyi Biotec, Sunnyvale, CA, USA). Briefly, cocultured cells were collected, washed twice with buffer (PBS supplemented with 2 mM EDTA and 0.5% bovine serum albumin-BSA), and incubated with CD11b microbeads for 15 min at 4°C. Washed and resuspended cells were applied to MACS column, which retained CD11b^+^ cells and allowed negative cells (adipocytes) to pass through. The column was then removed from the separator and placed on a suitable collection tube. Appropriate amounts of column buffer were pipetted onto the column to flush out positive cells (macrophages) using a plunger supplier with the column. This method resulted in 90% to 95% pure CD11b^+^ cells, as evaluated by flow cytometry. 

### 2.10. Flow Cytometry (FACS) Analysis

3T3-L1 adipocytes and Raw264.7 macrophages treated with obesity-related factors (as described earlier) were gently trypsinized, washed twice in PBS, and incubated with Fc*γ* receptor-blocking antibodies (24G2) for 10 minutes on ice, then stained with phycoerythrin (PE) conjugated anti-4-1BB (eBioscience, San Diego, CA, USA), anti-4-1BBL (eBioscience), or anti-Rat IgG2_a,k_  (eBioscience), Golden Syrian Hamster IgG (eBioscience) and anti-CD11b (eBioscience) as a control to define the gate for adipocytes/macrophages. The cells were then washed with FACS buffer and analyzed on a FACSCalibur (BD Biosciences, San Jose, CA, USA) with CellQuest software (BD Biosciences). Number in the graphs indicates the percentages of positive cells.

### 2.11. Western Blot Analysis

3T3-L1 adipocytes were plated at 1 × 10^6^ cells/well in 6-well plates and incubated with 3E1 (1 *μ*g/mL) or rat IgG for 3 h. Raw264.7 macrophages were plated at 1 × 10^6^ cells/well in 6-well plates coated with r4-1BB Fc or human IgG for 1 h. The 3E1-treated adipocytes and r4-1BB Fc-treated macrophages were rinsed with PBS, resuspended by scraping in lysis buffer (10 mM Tris-HCl, 10 mM NaCl, 0.1 mM EDTA, 50 mM NaF, 10 mM Na_4_P_2_O_7_, 1 mM MgCl_2_, 0.5% deoxycholate, 1% IGEPAL, and protease inhibitors cocktail), and centrifuged at 3000 rpm for 5 minutes. Samples containing 10–30 *μ*g of total protein were subjected to western blot analysis using polyclonal antibodies to phosphorylated IKK (I kappa B kinase alpha/beta; p-IKK *α*/*β*, Ser180/Ser181), total IKK*β*, p-p38 MAPK (mitogen-activated-protein kinase), p-JNK (c-Jun amino-terminal kinase), total JNK, p-Akt (protein kinase B; Ser473), total Akt (Cell Signaling, Danvers, MA, USA), and I*κ*B*α* (inhibitor of nuclear factor-*κ*B alpha; Santa Cruz Biotechnology, Santa Cruz, CA, USA), and *β*-actin (Sigma).

### 2.12. Statistical Analysis

Results are presented as means ± SEM of three independent performed in duplicate. Statistical comparisons were performed using Student's *t*-test or ANOVA with Duncan's multiple-range test. Differences were considered to be significant at *P* < 0.05.

## 3. Results

### 3.1. Expression of 4-1BB and 4-1BBL in Adipocytes/Macrophages and Adipose Tissue

**Figure 1:**
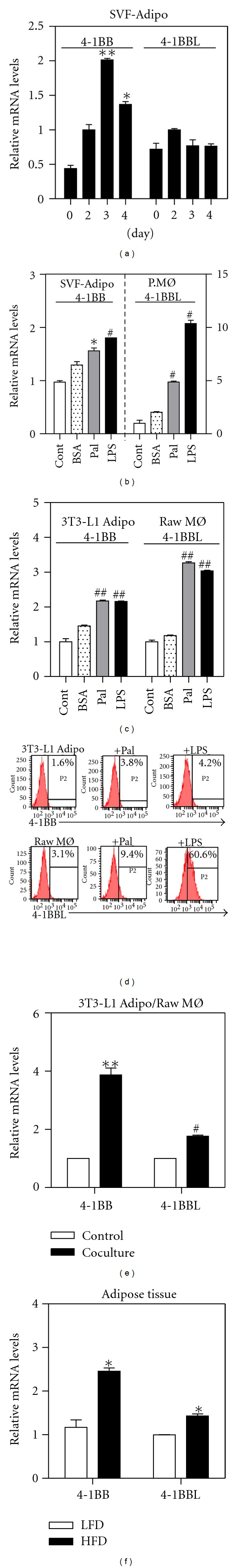
4-1BB and 4-1BBL expression is upregulated by obesity-related factors in adipocytes and macrophages. 4-1BB and 4-1BBL mRNA expression in SVF-derived adipocytes (a) during adipogenesis. SVF-derived confluent preadipocytes (day 0) were differentiated into adipocytes (days 2–4), as described in Section 2. 4-1BB and 4-1BBL mRNA levels in SVF-derived adipocytes and peritoneal macrophages treated with obesity-related factor (250 *μ*M Pal, 100 ng/mL LPS) for 24 and 4 h (b). 4-1BB/4-1BBL mRNA (c) and protein expression (d) in 3T3-L1 adipocytes and Raw264.7 macrophages treated with obesity-related factor (250 *μ*M Pal, 100 ng/mL LPS) for 24 h and 4 h. mRNA was measured by qRT-PCR, and protein levels were detected by FACS. 4-1BB and 4-1BBL mRNA levels in 3T3-L1 adipocytes/Raw264.7 macrophages cocultured for 24 h (e), control indicates mixed adipocytes/macrophages, which were cultured separately for 24 h and mixed after harvest, and in the epididymal adipose tissue (f) of mice fed a high-fat diet (HFD) or low-fat diet (LFD) (*n* = 4 mice per group). Levels of mRNA were estimated by qRT-PCR. Pal, palmitic acid; Adipo, adipocytes; P.MØ, peritoneal macrophages; Raw MØ, Raw264.7 macrophages. Data are the mean ± SEM of three independent experiments performed in duplicate. *P < 0.05; **P < 0.01; ^#^ *P* < 0.005; ^##^ *P* < 0.001 (compared with control).

We first measured expression of 4-1BB during adipogenesis at the mRNA level using qRT-PCR. We found that levels of 4-1BB transcripts were greater in SVF-derived adipocytes after differentiation (Figure 1(a)). Importantly, obesity-related substances such as palmitic acid and LPS significantly upregulated levels of 4-1BB transcripts in SVF-derived adipocytes and 4-1BBL transcripts in peritoneal macrophages (Figure 1(b)). The upregulation of the transcripts is confirmed in 3T3-L1 adipocytes and/or Raw264.7 macrophages (Figure 1(c)). FACS analysis also revealed that 4-1BB protein on 3T3-L1 adipocytes and 4-1BBL protein on Raw264.7 macrophages (Figure 1(d)) were increased by these obesity-related factors. In addition, 4-1BB and 4-1BBL transcripts increased in cocultured adipocytes/macrophages (Figure 1(e)), as well as in the epididymal adipose tissue of obese mice fed an HFD (Figure 1(f)).

### 3.2. Release of Inflammatory Cytokines and Activation of Inflammatory Signaling Molecules by 4-1BB and 4-1BBL Stimulation in Adipocytes and Macrophages, Respectively

**Figure 2:**
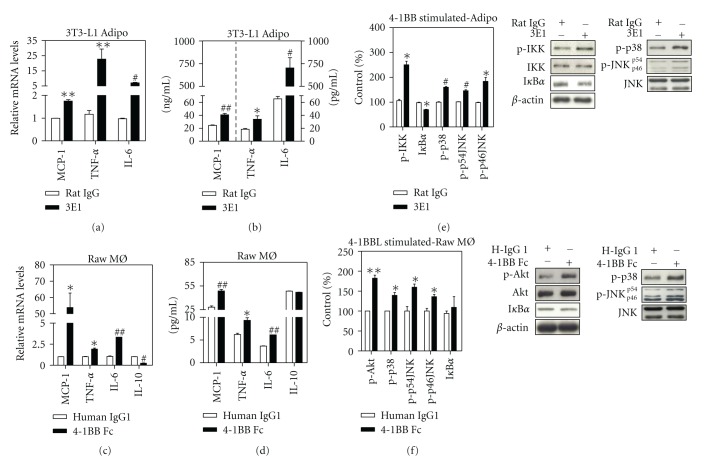
4-1BB or 4-1BBL stimulation enhances release of various inflammatory cytokines from adipocytes or macrophages. 3T3-L1 adipocytes and Raw264.7 macrophages were treated with 1 *μ*g/mL 3E1 and 100 ng/mL r4-1BB Fc, respectively, for 48–24 h. MCP-1, TNF-*α*, IL-6, and IL-10 transcripts and proteins were then measured in the 3T3-L1 adipocytes (a-b) and Raw264.7 macrophages (c-d). The phosphorylation of IKK*α*/*β* (p-IKK*α*/*β*)/IKK*β*, I*κ*B*α*, p-p38, p-JNK/JNK, pAkt/Akt and *β*-actin were measured by western blotting. 3T3-L1 adipocytes were incubated with 1*μ*g/mL 3E1 for 3 h (e), and Raw264.7 macrophages with 100 ng/mL r4-1BB Fc for 1 h (f). Results of densitometry were showed as percentage of control. Data are the mean ± SEM of three independent experiments performed in duplicate. *P < 0.05; **P < 0.01; ^#^ *P* < 0.005; ^##^ *P* < 0.001 (compared with control).

To examine whether 4-1BB on adipocytes or 4-1BBL on macrophages provide an inflammatory signal, we treated each cell type with agonists that specifically stimulate these molecules; 3T3-L1 adipocytes were treated with an agonistic 4-1BB antibody (3E1) for 48 h, and Raw264.7 macrophages with r4-1BB-Fc for 24 h and we then measured levels of inflammatory cytokines in the respective cells. Both 4-1BB stimulation of adipocytes and 4-1BBL stimulation of macrophages markedly increased the production of proinflammatory cytokines such as MCP-1, TNF-*α*, and IL-6 at the mRNA (Figures 2(a), and 2(c)) and protein levels (Figures 2(b) and 2(d)). Adiponectin secretion from adipocytes was not altered by 4-1BB stimulation (data not shown). 4-1BBL stimulation of macrophages decreased the transcripts of IL-10 (Figure 2(c)), but no change was observed in IL-10 protein release (Figure 2(d)).

 To understand the molecular mechanisms by which 4-1BB and/or 4-1BBL activate inflammatory signaling in adipocytes and/or macrophages, we examined the effects of 4-1BB/4-1BBL stimulation on intracellular signaling molecules. Stimulation of 4-1BB on adipocytes increased the phosphorylation of p38 MAPK and JNK as well as the phosphorylation of IKK, the upstream molecule of NF-*κ*B, and induced I*κ*B*α* degradation (Figure 2(e)), while stimulation of 4-1BBL increased phosphorylation of Akt and p38 MAPK, and JNK (Figure 2(f)), but had no effect on degradation of I*κ*B*α* protein (Figure 2(f)) and phosphorylation of IKK (data not shown). Consistent with previous reports [20, 21], 4-1BBL signaling not only activated p38 MAPK but also induced Akt activation in macrophages, leading increased inflammatory cytokines expression.

### 3.3. Release of Inflammatory Cytokines in a Contact Coculture System

**Figure 3:**
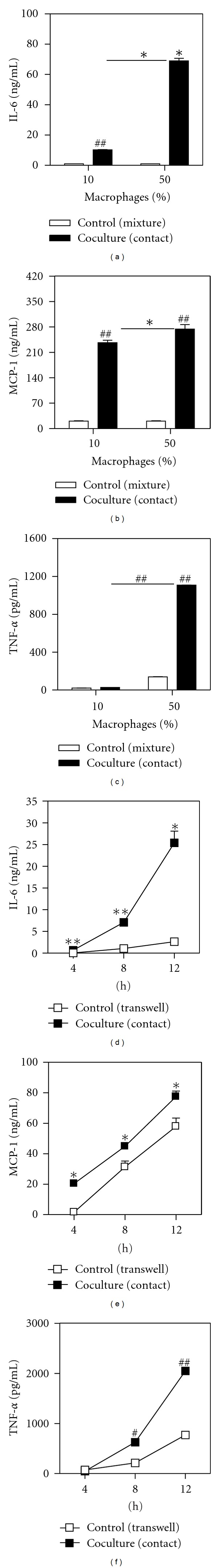
Release of inflammatory cytokines is enhanced in cocultures of 3T3-L1 adipocytes and Raw264.7 macrophages. Release of cytokines (IL-6, MCP-1, and TNF-*α*) from 3T3-L1 adipocytes cocultured with Raw264.7 macrophages (10% or 50%) for 24 h (a–c). Control indicates mixed adipocytes/macrophages, which were cultured separately for 24 h and mixed after harvest. Release of cytokines (IL-6, MCP-1, and TNF-*α*) from the contact coculture with 50% Raw264.7 macrophages or transwell system for indicated times (4 h, 8 h, and 12 h) (d–f). Cell-free supernatants were collected, and concentrations of these inflammatory cytokines were determined by ELISA. Data are the mean ± SEM of three independent experiments performed in duplicate. *P < 0.05; **P < 0.01; ^#^ *P* < 0.005; ^##^ *P* < 0.001 (compared with control).

Because 4-1BB/4-1BBL stimulation enhanced the release of inflammatory cytokines from adipocytes and/or macrophages, respectively, we investigated whether cell-cell interaction via surface molecules, presumably 4-1BB/4-1BBL, has a role in initiating and triggering inflammatory responses. We first cocultured 3T3-L1 adipocytes and Raw264.7 macrophages in a direct contact system and found that the production of inflammatory cytokines IL-6, MCP-1, and TNF-*α* was correlated with the number of macrophages in the culture (Figures 3(a)–3(c)) and increased with time (Figures 3(d)–3(f)). 

### 3.4. Effect of Disruption of the Interaction between 4-1BB and 4-1BBL on Release of Inflammatory Cytokines in a Contact Coculture System

**Figure 4:**
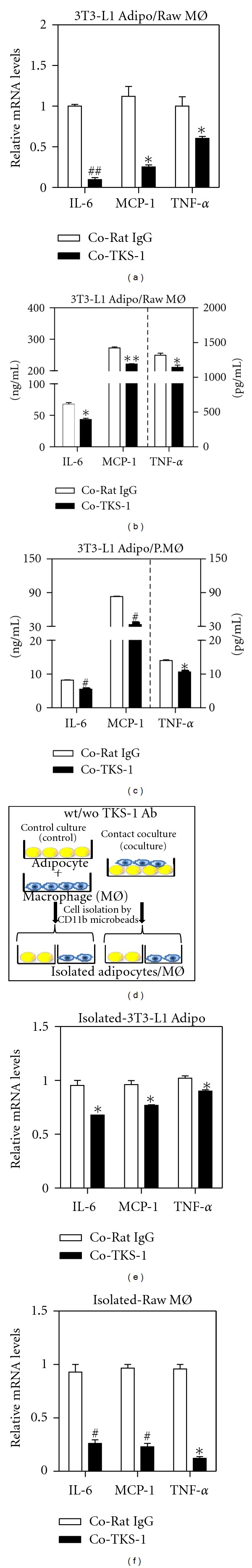
Disruption of the 4-1BB/4-1BBL interaction suppresses expression of inflammatory cytokines in direct contact cocultures. Raw264.7 macrophages were seeded onto 3T3-L1 adipocytes with/without pretreated with neutralizing anti-4-1BBL antibody (TKS-1) or Rat IgG (5 *μ*g/mL) in serum-free medium for 24 h. Total RNAs were isolated and analyzed the level of IL-6, MCP-1, and TNF-*α* by qRT-PCR (a). The protein levels of IL-6, MCP-1, and TNF-*α* in cell-free supernatants were collected from 3T3-L1 adipocytes and Raw264.7 macrophages coculture (b) and from cocultures of 3T3-L1 adipocytes and peritoneal macrophages (c). Illustration of coculture system with adipocytes/macrophages (d). After 24 h coculture of Raw264.7 macrophages and 3T3-L1 adipocytes, these cells were separated using the CD11b MicroBead system. Levels of inflammatory cytokine mRNAs were detected in the adipocytes (e) and macrophages (f). Co, coculture. Data are the mean ± SEM of three independent experiments performed in duplicate. *P < 0.05; **P < 0.01; ^#^ *P* < 0.005; ^##^ *P* < 0.001 (compared with rat IgG-treated cells).

To test whether the 4-1BB/4-1BBL-mediated interaction between adipocytes and macrophages participates in the inflammatory response in the contact cocultured 3T3-L1 adipocytes/Raw264.7 macrophages, we blocked the interaction using a neutralizing antibody (TKS-1). The neutralizing monoclonal antibody reacts specifically with mouse 4-1BBL, by which 4-1BBL cannot bind to 4-1BB receptor and can interrupt the interaction between 4-1BBL and 4-1BB. Hence, both 4-1BB-mediated signal in adipocytes and 4-1BBL-mediated signal in macrophages can be blunted by TKS-1 treatment. We found that treatment with TKS-1 significantly reduced levels of IL-6, MCP-1, and TNF-*α* mRNA in the contact cocultured adipocytes/macrophages (Figure 4(a)). The reduction in the expression of these inflammatory cytokines was confirmed at the protein level (Figure 4(b)). Moreover, we also found that disruption of the interaction between 4-1BB and 4-1BBL reduced the release of inflammatory cytokines from peritoneal macrophages cocultured with adipocytes (Figure 4(c)). To examine the relative contributions of 4-1BB and 4-1BB signaling to inflammatory gene expression in the cocultured adipocytes/macrophages, we separated the macrophages from the adipocytes and measured levels of inflammatory cytokine transcripts in the two types of cell (Figure 4(d)). The neutralizing antibody significantly reduced the increase in levels of IL-6, MCP-1, and TNF-*α* mRNAs in the adipocytes as well as in the macrophages (Figures 4(e) and 4(f)).

## 4. Discussion

Obesity-induced adipose inflammation is characterized by recruitment of macrophages into adipose tissue, and the macrophages are an important source of inflammatory responses. Cell-cell contact between adipocytes and macrophages is considered to be important for triggering inflammatory pathways in adipose tissue [5, 6], although it is unclear which molecules are involved. Recent studies have shown that the engagement of co-stimulatory receptor 4-1BB with its ligand 4-1BBL through cell-cell contact modulates various inflammatory responses [13, 14, 16]. In previous work, we found that 4-1BB deficiency reduced adipose inflammation by decreasing macrophage recruitment and the release of inflammatory cytokines [17]. Based on these findings, we hypothesized that 4-1BB/4-1BBL-mediated cell-cell interaction between adipose cells and macrophages might be important in the onset and/or maintenance of obesity-induced adipose inflammation. Interestingly, 4-1BB transcripts in adipocytes and 4-1BBL transcripts in macrophages were markedly upregulated by obesity-related factors (e.g., FFA and LPS), and their expression was also strongly increased in contact cocultured adipocytes/macrophages. Moreover, the upregulation of these molecules was accompanied by enhanced release of inflammatory cytokines from the cells. These findings together with the upregulation in obese adipose tissue and the reduction of adipose inflammation in 4-1BB-deficient obese mice [17] suggest that 4-1BB and 4-1BBL participate in the onset and/or promotion of adipocytes/macrophage-induced inflammatory responses. 

**Figure 5:**
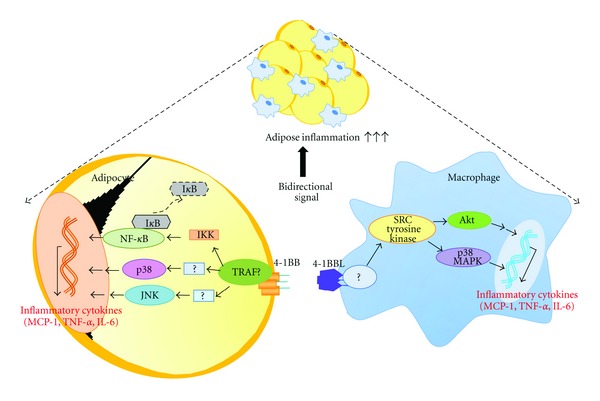
Schematic representation of the bidirectional signal transduction induced by 4-1BB/4-1BBL-mediated interaction between adipocytes and macrophages. The release of inflammatory cytokines (MCP-1, TNF-*α*, and IL-6) in response to the bidirectional signaling appears to participate in adipose inflammation.

In order to see whether 4-1BB on adipocytes or 4-1BBL on macrophages was responsible for the inflammatory signals that triggered inflammatory responses, we stimulated the cells with agonists which bind specifically to either 4-1BB or 4-1BBL. We found, for the first time, that stimulation of 4-1BB on adipocytes markedly increased the release of inflammatory cytokines MCP-1, TNF-*α*, and IL-6. Stimulation of 4-1BBL-mediated reverse signaling, which is known to activate macrophages [22], also increased levels of inflammatory cytokines. Recent evidence indicates that 4-1BB signaling results in activation of the MAPK/NF-*κ*B pathway, which is TNF receptor-associated factor (TRAF)-2-dependent [23] in lymphocytes [24]. In adipocytes, we found that stimulation of 4-1BB activated p38 MAPK, JNK, IKK and induced I*κ*B*α* protein degradation. On the other hand, stimulation of 4-1BBL led to activation of inflammatory signaling molecules such as Akt and p38 MAPK in macrophages, which is consistent with previous studies [10, 20, 21]. More importantly, we found that treatment with a 4-1BBL neutralizing antibody reduced release of inflammatory cytokines in cocultures at both the mRNA and protein level. These findings together suggest that the 4-1BB/4-1BBL-mediated interaction between adipocytes and macrophages triggers bidirectional inflammatory signaling and is a potent inducer of inflammatory responses in obese adipose tissue (Figure 5). 

Interestingly, disruption of the 4-1BB/4-1BBL interaction did not completely suppress release of inflammatory cytokines from cocultured adipocytes and macrophages. This may be due to the presence of other cell surface molecules which participate in cell-cell interactions and mediate inflammatory responses. Indeed, adipocytes and macrophages express many inflammatory receptors and ligands on their surfaces [6, 25, 26]. For example, CD40 and herpes virus entry mediator (HVEM), which are expressed on adipocytes, are considered to be mediators of contact-dependent signaling of macrophages, and ablation of these receptors reduces obesity-induced inflammatory responses [26–30]. Thus it is conceivable that other receptors and ligands in addition to 4-1BB and 4-1BBL are also involved in the interaction between adipocytes and macrophages that leads to the initiation and maintenance of inflammatory responses. 

In conclusion, we have demonstrated for the first time that the contact-dependent interaction between adipocytes and macrophages mediated by 4-1BB and 4-1BBL, which generates bidirectional signals, plays a crucial role in the release of adipose inflammatory cytokines from these cells. 4-1BB and 4-1BBL, along with other molecules involved in cell-cell interaction between adipocytes and macrophages, may be valuable targets for preventing obesity-induced adipose inflammation.

## Figures and Tables

**Table 1:** Sequences of mouse primers used for qRT-PCR analysis.

Gene	Forward primer sequence	Reverse primer sequence
4-1BB	CTCTGTGCTCAAATGGATCAGGAA	TGTGGACATCGGCAGCTACAA
4-1BBL	CCTGTGTTCGCCAAGCTACTG	CGGGACTGTCTACCACCAACTC
MCP-1	GCATCCACGTGTTGGCTCA	CTCCAGCCTACTCATTGGGATCA
TNF-*α*	AAGCCTGTAGCCCACGTCGTA	GGCACCACTAGTTGGTTGTCTTTG
IL-6	CCACTTCACAAGTCGGAGGCTTA	GCAAGTGCATCATCGTTGTTCATAC
36B4	TGTGTGTCTGCAGATCGGGTAC	CTTTGGCGGGATTAGTCGAAG
*β*-actin	CATCCGTAAAGACCTCTATGCCAAC	ATGGAGCCACCGATCCACA
